# Development and validation of an instrument to measure the professional’s knowledge of dispensing medication (CDM-51) in community pharmacies

**DOI:** 10.1371/journal.pone.0229855

**Published:** 2020-03-03

**Authors:** Ana Maria Rosa Freato Gonçalves, Marília Silveira Almeida Campos, Andrea Bernardes, Carmem Silvia Gabriel, Leonardo Régis Leira Pereira

**Affiliations:** 1 Pharmaceutical Assistance and Clinical Pharmacy Research Center, Department of Pharmaceutical Sciences, School of Pharmaceutical Sciences of Ribeirão Preto, University of São Paulo (USP), Ribeirão Preto, Brazil; 2 Department of General and Specialized Nursing, College of Nursing, University of São Paulo, Ribeirão Preto, Brazil; University of South Australia, AUSTRALIA

## Abstract

Medication dispensing performed without the necessary information on proper use can result in harmful effects to the individual, and therefore providing this service with quality for the users is necessary to promote the rational use of medication; however, in a developing country this activity is performed largely by unqualified people and in an inappropriate way. This study aims to develop and validate a study instrument that measures the knowledge of medication dispensing for the professionals involved in this practice (pharmacist, pharmacy technician in the pharmacy, and clerk/assistant). The study has methodological design and is characterized by the development and validation of an instrument to measure the knowledge of dispensation. A questionnaire denominated CDM-51 was elaborated and divided in two parts: the first collects the socio-demographic characteristics of the participants, and the second has 51 questions to assess the knowledge construct regarding dispensation. The validity of content was realized through the evaluation by seven experts regarding the relevance and clarity of the items. A pretest and main validation study with 30 and 79 pharmacy professionals respectively, from the city of Ribeirão Preto in the Brazilian state of São Paulo were carried out, and questions presented to the respondents were corrected. The analysis of the internal consistency of the KR-20 (Kuder-Richardson) was 0.837, and validity construct evidence was found (p value: 0.001) that participants with formal education have greater knowledge of medication dispensing. This work contributes to increasing the quality of services provided by dispensing pharmacies and points out the importance of training for formal education to perform this service, thus promoting the rational use of medication.

## Introduction

The community pharmacy is an easily accessible public health facility where the main service offered is medication dispensing [1–2]. However, medication dispensing performed without the necessary information on proper use can result in harmful effects to the individual [3–4], and therefore providing this service with quality for the users is necessary to promote the rational use of medication [5–7].

To carry out proper dispensation, the professional must have sufficient knowledge to guide the patient on the correct use of the medication, of interaction with other medication and foods, recognition of potential adverse reactions, and conditions of product preservation. Formal education may be important for professionals to appropriate this knowledge [2,8–9]. In addition, professionals must know the legislation related to the medication dispensing process, which aims to guarantee the quality thereof.

In developing countries such as Brazil, medication dispensing is performed under pharmacist supervision at community pharmacies. However, this activity is commonly carried out by professionals who are not pharmacists or technicians in the pharmacy [10–11]. In other words, in Brazilian community pharmacies it is common to find professionals without formal professional qualifications dispensing medication, because the presence of a pharmacy technician is not required in community pharmacies. [12]. In this context, it is important to highlight that this fact can compromise the quality of the service provided to the population [2,8,13].

When analyzing pharmacy services from Donabedian’s (1966) viewpoint, it is important to evaluate these services in view of their structure, and material, human, and organizational resources [14]. Thus, we can observe that the evaluation of the personnel who develop the medication dispensing in pharmacies can be a tool to determine the quality of this service provided; and the knowledge of medication dispensing from the professionals involved in this practice is a way of estimating the quality of such service.

In this sense, a number of studies have evaluated the knowledge of medication dispensing of the professionals involved in this practice [9,8,11,15–17], and studies show that medication dispensing by untrained professionals corroborates the irrational use of the medication [8,18–19]. Thus, the importance of conducting such studies is highlighted, especially in developing countries where formal training of these professionals is not standardized.

In view of the methodological robustness and reliability of the results obtained, the use of validated instruments is very important. However, no validated instrument has been found in the literature to evaluate knowledge about medication dispensing. Thus, the proposal of this study was the construction and validation of an instrument capable of measuring the knowledge of medication dispensing by professionals working in community pharmacies.

## Method

### Development of the CDM-51

In order to provide robustness to the methods used, the questionnaire was prepared and validated following the recommendations of Pasquali (2010); Hulley (2003); and Lobiondo-Wood, Haber (2001) [20–22].

Initially, a review was carried out in scientific papers and books to identify relevant topics to measure the knowledge of medication dispensing. At the same time, interviews were conducted using a semi-structured instrument [23] with seven experienced pharmacists in the medication dispensing area, and two doctors and one master in the area of pharmaceutical assistance.

After this review, a structured questionnaire containing two parts was created. The first characterizes the participant’s profile regarding the level of schooling, age, gender, time spent in the medication dispensing area, and the data source used by the participant for research during medication dispensing. These variables provide support for the evaluation of possible correlations between the knowledge regarding dispensation and the profile of the interviewee, considering whether the interviewee is a clerk, pharmacy technician or pharmacist.

The second part has objective questions that are intended to evaluate the knowledge construct of medication dispensing based on the following laws and regulations:

- Attitudes allowed in the pharmaceutical environment [24–26];
- Dispensing of medication subject to special control [27–28];
- Dispensing of generic medication [29];
- Dispensing of antimicrobials [30];
- Dispensing of medication exempt from medical prescription [31];

In addition, we also included items on the medication dispensing used for diseases of high prevalence (systemic arterial hypertension, diabetes mellitus, dyslipidemias, asthma, rheumatoid arthritis, depression, and epilepsy) [32]. The domain of these sources is a determinant factor to dispense medication correctly, i.e., all professionals involved in the dispensation: clerks, pharmacy technicians, and pharmacists must master this legislation [6,24,33].

The first version of the questionnaire had 20 items and was submitted to the evaluation of the clarity and pertinence of each item by seven judges, all of them specialists in the medication dispensing area: six doctors and one master [34–36]. As a result of this process the experts suggested the inclusion of new items, thus a second version of the 51-item questionnaire was returned to the same judges, resulting in the third version of the questionnaire (51 items). It should be noted that a concordance percentage of at least 80% of the judges was adopted for each item.

In order to evaluate possible difficulties in responding to this questionnaire (cognitive properties), and also as a component of content validity, a pre-test was performed using a sample number of 30 [37] pharmacy employees from the city of Ribeirão Preto, in the state of São Paulo (SP) resulting in the latest version, named the Questionnaire for Assessment of Knowledge about Medication Dispensing (*Questionário para Avaliação do Conhecimento sobre Dispensação do Medicamento* CDM-51).

For analysis of the psychometric properties of the CDM-51, i.e., analysis of internal consistency, construct validation, and interpretability, this structured questionnaire was applied to a sample of 79 pharmacy employees and this stage was denominated as the “main validation study”.

### Validation of the CDM-51

#### Study population

Pharmacy employees from the municipality of Ribeirão Preto, Brazil who act in the process of medication dispensing, being pharmacists, pharmacy technicians, and clerks.

The municipality of Ribeirão Preto (SP) has 658,059 inhabitants [38] with approximately one community pharmacy for every 2,800 inhabitants.

The selection criteria for the sample were: employees who worked in the pharmacies visited that offered the service of dispensation of industrialized medication. The exclusion criteria were: pharmacies that were closed, or employees who were not present at the time of the visit by the researcher.

The pharmacies were visited until the sample number needed for the selected statistical analyzes was reached. It was not previously determined which pharmacies should be visited; data collection was carried out from June to October 2015. The researcher applying the CDM-51 ensured that the questionnaire was answered individually.

This work was submitted to the Ethics Committee in Research involving human beings of the Faculty of Pharmaceutical Sciences of Ribeirão Preto—FCFRP under the number CAE: 34.700.514.300.005.403.

#### Statistical analysis

The data obtained were tabulated using the Microsoft Office Excel^®^ program (Office 2013). Descriptive statistics were performed using absolute and relative frequency (%) for the categorical variables, as well as the mean and standard deviation, or median, minimum and maximum for the quantitative variables. Descriptive data were used to analyze the interpretability.

The Statistical Package for Social Sciences (SPSS) Inc., version 17.1.0, 2008 was used to perform the statistical analyzes. For internal consistency analysis the Kuder-Richardson test (KR-20) was calculated because it is a questionnaire whose answers are dichotomous; and in the validation of construct, variance analysis (ANOVA with a factor) was performed to evaluate if there is evidence that formal education provides a higher score in the CDM-51. The Bonferroni test was also used to evaluate in which group (pharmacist, clerk, or pharmacy technician) that difference is found.

### Results

**Fig 1:**
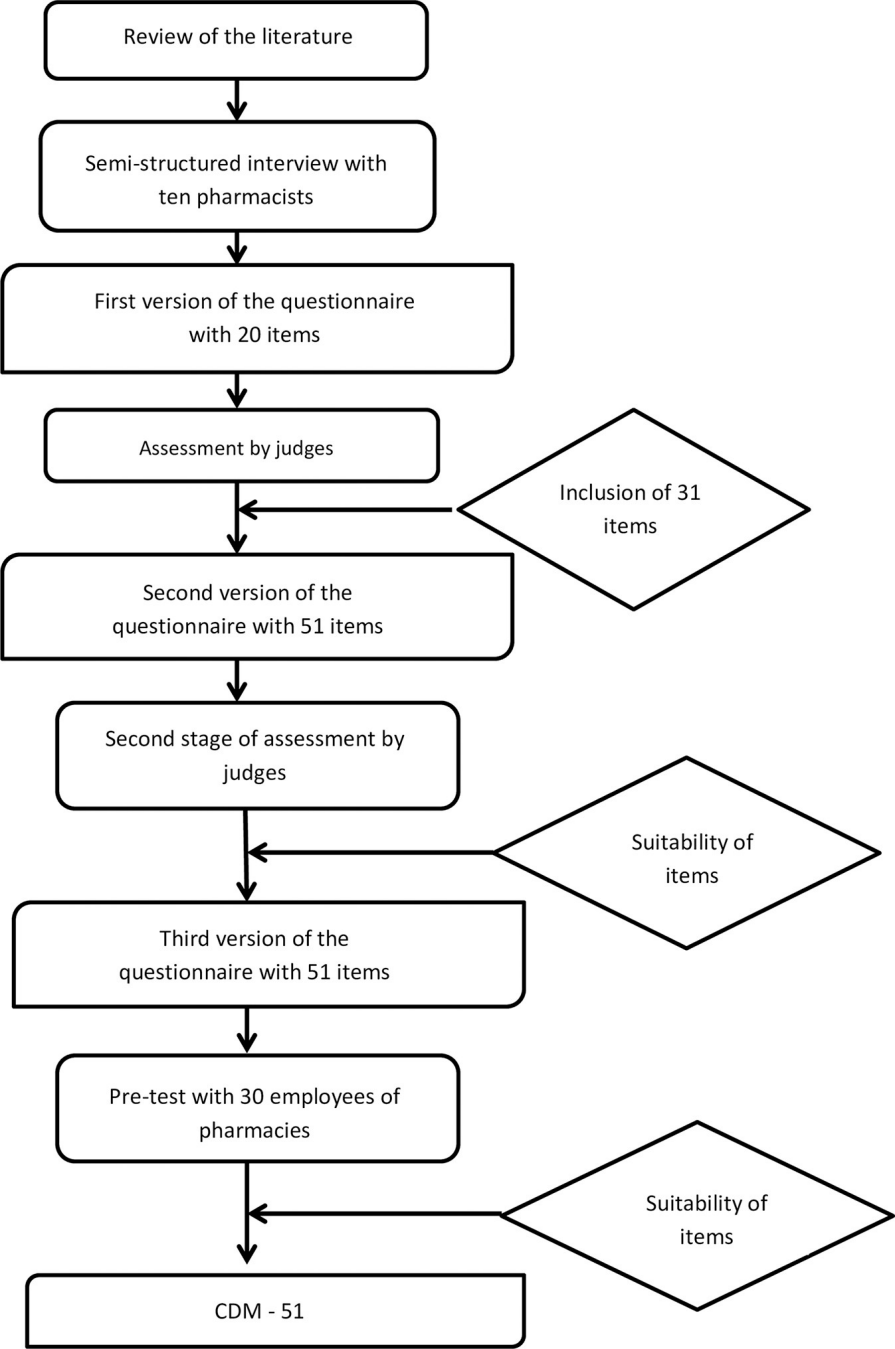
Summary of elaboration stages of the CDM-51.

The first version of the questionnaire was analyzed by the committee of judges, with 63.15% of the items considered pertinent and 42.1% considered clear. In addition, the judges pointed out suggestions for introducing new issues. After this analysis, the suggestions were evaluated and resulted in a new version with 51 items, which was forwarded to the seven judges, but only six responded to the new assessment. In the second analysis, 100% of the questions were assessed as pertinent, 82% were assessed as clear, and no suggestions for introducing new issues were presented. After this step, the third version of the instrument containing the same number of items was obtained (Fig 1).

The pretest population (N: 30) presented doubts when answering the questionnaire (Table 1), and modifications were performed, resulting in the final version of CDM-51 (S1 Appendix).

**Table 1:** CDM-51 items modified after the pre-test phase.

*Summary of the question*	*Difficulty encountered*	*Summary of the modified question*
The pharmacy can sell drug-related products.	Difficulties in defining the term ‘drug-related products’.	The pharmacy can sell products classified as drug-related products (Examples: syringe, needle).
The yellow prescription notification is intended for the dispensing of psychotropic drugs.	Double interpretation.	The blue prescription notification is intended for the dispensing of narcotic drugs.
Prescription of antiparkinson and anticonvulsant drugs may be valid for up to six months.	Double interpretation.	Antiparkson and anticonvulsant drug prescriptions may contain enough quantity for up to six months of treatment.
Glibenclamide 5 mg should be given with a glass of water half an hour before the main meals (breakfast, lunch and dinner), according to medical prescription.	Difficulties of understanding in discourse.	Glibenclamide 5 mg, when prescribed, should be given half an hour before the main meals (breakfast, lunch and dinner), with a glass of water.
Captopril 25 mg should be given 1–2 hours after meals.	Double interpretation.	Captopril 25 mg, when prescribed, should be given 1–2 hours after meals.
Hydrochlorothiazide 25 mg should be given with food.	Double interpretation.	Hydrochlorothiazide 25 mg, when prescribed, can be given on an empty stomach or with food.
Simvastatin may be given on awakening or at bedtime, according to medical prescription.	Difficulty of understanding in discourse.	Simvastatin, when prescribed, may be administered on awakening or at bedtime.
Insulins in use can be stored at room temperature (15 °C to 30 °C) or under refrigeration.	Double interpretation.	Insulins in use can be stored at room temperature (15 °C to 30 °C) for up to 30 days or under refrigeration (2 °C to 8 °C) for up to 3 months.

The CDM-51 has items with the following answer options: ‘true’, ‘false’, and ‘I do not know’; each correct answer assigns a score value of one and incorrect answers and/or marked as ‘I do not know’ are assigned a zero score, so the questionnaire score varies from 0 to 51 and the higher the value obtained, the greater the knowledge about medication dispensing.

#### Validation of the CDM-51

The CDM-51 was answered by 109 community pharmacy employees, and socioeconomic characteristics can be observed in Table 2. Most of the participating employees were pharmacists and of these the majority were women. The mean age found among the pharmacists participating in the main validation study was 35.63 (8.11) years, a value similar to that found for the other categories. The average time of training of pharmacists and pharmacy technicians respectively, was 10.45 (7.85) and 8.33 (6.38) years. The average time of experience of the clerks was the highest (12.26 SD: 10.31). Half of the pharmacists have a postgraduate course, but only 6.52% of these professionals present postgraduate studies in the area of medication dispensing. The clerks have on average 12.33 (3.55) years of study.

**Table 2:** Socio-demographic characteristics of the participants in pre-test phase and main validation study.

*Variables*	*Pharmacist*	*Pharmacy technician*	*Clerk*
	**Pre-test phase N = 30**		
Total of participating employees N (%)	14 (46.66%)	2 (0.68%)	14 (46.66%)
Gender N (%) Women	9 (64.28%)	2 (100%)	8 (57.1%)
Age in years: Mean (SD)	39.93 (11.48)	34 (7.07)	40.92 (9.37)
Experience time (years): Mean (SD)	13.57 (9.52)	3*	17.59 (8.58)
Graduation time (years): Mean (SD)	15.35 (12.11)	8.5 (0.7)	-
Pharmacists with postgraduate courses N (%)	7 (50%)	-	-
Post-graduation in drug dispensing	0	-	-
Years of study: Mean (SD)	11.30 (4.36)	-	-
	**Main study N = 79**		
Total of participating employees N (%)	46 (58.22%)	18 (22.78%)	15 (18.98%)
Gender N (%) Women	34 (73.91%)	12 (66.66%)	6 (60%)
Age in years: Mean (SD)	35.36 (8.11)	32.11 (9.90)	35.9 (7.47)
Experience time (years): Mean (SD)	10.85 (7.45)	10.66 (10.66)	12.26 (10.31)
Graduation time (years): Mean (SD)	11.21 (7.94)	8.33 (6.38)	-
Pharmacists with postgraduate courses N (%)	23 (50%)	-	-
Post-graduation in drug dispensing	3 (6.52%)	-	-
Years of study: Mean (SD)	12.33 (3.55)	-	-

In the internal consistency analysis, a KR-20 value of 0.837 was found through the data obtained in the main validation study, and when each CDM-51 question was withdrawn, the KR-20 value was not lower than 0.800.

Regarding construct validity, the ANOVA test provided evidence that there is a difference between the means obtained in the questionnaire score among the professionals studied. Thus the Bonferroni test was performed, which showed evidence that formal education in the pharmacy undergraduate modality allows a higher average score in the questionnaire (Table 3). However, no evidence was found that the technical modality in pharmacy, combined with practice, allows a higher average score in relation to the clerks.

**Table 3:** Differences between mean scores obtained in the CDM-51 by professional categories.

*Professionals*	*Mean Difference*	*p value*	*95% Confidence Interval of the mean difference*
*Pharmacist/Clerk*	9.033	<0.001	4.09–13.97
*Pharmacist/Pharmacy technician*	11.083	0.001	3.76–18.40
*Pharmacy technician /Clerk*	-2.050	1.000	-10.30–6.20

When analyzing the interpretability of the questionnaire, i.e., evaluating the differences found when using the instrument in different groups, it is possible to observe in Table 4 that there were differences between the means of the scores obtained in the CDM-51 by the different groups evaluated.

**Table 4:** Scores obtained in the CDM-51 by professional categories.

*Groups*	*Sample size*	*Mean*	*Standard Deviation*	*95% Confidence Interval of the mean*	*Minimum*	*Maximum*
*Pharmacist*	46	36.32	6.09	34.46–38.21	23	49
*Clerk*	15	27.30	4.78	23.88–30.72	19	37
*Pharmacy technician*	4	25.25	5.90	15.85–34.65	17	39

## Discussion

Assessing the quality of the medication dispensing service that the community pharmacy offers to the population is important in view of the impact of this service on the rational use of medication, since it creates opportunities for users to be informed of information relevant to the use of the medication [7,39–40]. Another relevant factor to be highlighted is that after the industrialized production of the medication, the pharmacist moved away from activities related to patient care, including medication dispensing, and this fact in Brazil was highlighted after the publication of Law 5991/73 that allowed the opening of pharmacies by laymen [24, 41]. In this context, the CDM-51 offers an opportunity to carry out this evaluation by measuring the knowledge of the professionals involved in this practice regarding medication dispensing.

It should be noted that the undergraduate course in pharmacy includes disciplines that aim to enable the pharmaceutical professional to carry out medication dispensing, as well as supervise the activities involved in dispensing the medication carried out by other professionals who work in the pharmacy (clerk and pharmacy technician). Therefore all the legislation contained in the CDM-51, as well as disciplines aimed at the rational use of the medication are considered in the curriculum of this professional [42–43].

The United States of America (USA), as well as other developed countries, presents a technical course in pharmacy, and the pharmacy technician is the only person qualified to work under the supervision of the pharmacist in pharmacies in the USA [44]. The Pharmacy Technician course (in the USA), as well as in Brazil, covers basic disciplines of anatomy, physiology, and pharmacology [44–45]. El Hajji et al. (2015) highlighted the importance of this professional to clinical pharmacy under the supervision of a clinical pharmacist [46].

The clerk must be trained by the pharmacist to act in the dispensation process under the responsibility of the pharmacist [25]. However, in underdeveloped countries such as Brazil, the legislation does not explain how this training should be, nor does it indicate that this professional must have formal education for medication dispensing. Therefore, the items present in the CDM-51 to measure knowledge about medication dispensing should be known by both the pharmacist and by the other professionals who work in this activity under the supervision of the pharmacist. In this way, it underlines the importance of the CDM-51 as a tool for the development of studies that assess the quality of dispensation through the knowledge of the employees involved.

In relation to the CDM-51 validation process, in the content validity analysis, Streiner and Norman (2008) recommend that the judges assess in one phase [36]; however, in view of the high number of suggestions for inclusion of new items, it was decided to carry out two phases of this analysis with the objective that such added items should also be evaluated by the judges’ committee.

Regarding the analysis of the internal consistency of the instrument, some authors recommend that the value of the total KR-20 and of the attributes of the questionnaire be greater than or equal to 0.70 [47–49], while other authors recommend that this value be within the range of 0.70 to 0.90 [35]. Based on this evidence, it can be observed that the questionnaire shows good internal consistency, since the KR-20 value of CDM-51 was 0.837. It is important to emphasize that having a good internal consistency means that the items of the instrument have covariates at similar points and this fact denotes evidence that the items measure a set of the same construct [50].

In the analysis of interpretability, it is possible to observe that there were differences in the descriptive statistics of the score obtained in the CDM-51 among the professional categories evaluated (Table 4), which shows that the questionnaire has the capacity to show a quantitative score in a qualitative attribute. In addition, it is possible to analyze construct validity by testing the instrument’s hypothesis [35]; evidence was found that pharmacists obtained a higher score than other professionals (Table 3), i.e., there is evidence that the pharmacists participating in this study obtained a higher score than other pharmacy employees. In this way, it is possible to observe the construct validity of the CDM-51 instrument.

We acknowledge some limitations of this study. The first limitation refers to the reduced number of pharmacy technicians (N: 18) who answered the questionnaire, since this fact may have driven the result that no evidence was found that the technical modality in pharmacy allows a higher average score with relation to clerks. However, it is important to point out that it is not common to find these professionals working in pharmaceutical establishments, since in Brazilian pharmacies as well as in other developing countries, training through the formal education of non-pharmaceutical labor is not mandatory [51]. The other limitation was the use of laws as support for some items of the CDM-51, since such laws, besides being contextualized to the Brazilian scenario, are subject to change; however the use of such laws was essential for the elaboration of the CDM-51 since the mastery of these laws is required for medication dispensing. For the generalization of CDM-51 in other countries, it is possible provided that a cross-cultural adaptation is carried out, taking into account the context of the country in question.

In view of these facts, it is possible to observe evidence that the CDM-51 has content validity, internal consistency, and construct validity. Thus, this instrument is able to measure the knowledge about medication dispensing of pharmacists, pharmacy technicians, and clerks working in Brazilian community pharmacies. Furthermore, through the CDM-51 it is possible to evaluate the workforce present in community pharmacies in order to identify alternatives to improve the dispensation service offered, and in this way, contribute to the increase in the quality of medication dispensing services.

## Conclusion

Considering the proposed objectives, the CDM-51 presents evidence that it has content validity, internal consistency, and construct validity.

## Supporting information

S1 Appendix (PDF)

S1 Data (PDF)
