# Supplementary material for: Development and validation of an instrument to measure the professional’s knowledge of dispensing medication (CDM-51) in community pharmacies
Source: PLoS One. 2020 Mar 3;15(3):e0229855. doi: 10.1371/journal.pone.0229855 (PMC7053717; doi:10.1371/journal.pone.0229855)
Supplement: S1 Appendix — (PDF) [file pone.0229855.s001.pdf]

1 Appendix A: Answers to the CDM – 51

Questions	Answer (T/F*)	Reference
Theme: Attitudes permitted in the pharmaceutical environment.		
Blood pressure and body temperature measurements are the physiological parameters permitted to be performed at the pharmacy.	T	Article 69°, paragraph 1°, of Directors' Collegiate Resolution (RDC) n°44/2009.[1]
The pharmacy can sell products classified as correlates (examples: syringe, needle).	T	Article 5°, paragraph 1°, of Law n° 5991/1973.[2]
Soft drinks (carbonated beverages based on guarana, cola, citrus, etc.) are classified as correlates in a pharmacy.	F	Article 4, clause IV, of Law 5991/1973.[2]
The sale of medication for prophylactic use in a pharmacy is permitted.	T	Article 4°, clause II, Law 5991/1973.[2]
Dispensation of an illegible prescription is only permitted when it is a medication of continuous use.	F	Article 35, line “a”, of Law n° 5991/1973.[2]
A pharmacy that does not have a complementary medication service may dispense complementary medication when it has another branch that does have this service.	F	Article 50° of RDC 44/2009.[1]
Only pharmacies that are physically open to the public, i.e., those that work at a fixed address and have face-to-face services, can offer the sale of medication via the Internet.	T	Article 52, subsection I, of RDC 44/2009.[1]
The pharmaceutical care service aims to prevent, detect, and solve problems related to medications, as well as the diagnosis of diseases of less complexity, and can only be performed by the pharmacist.	F	Article 66, subsection I, of RDC 44/2009.[1]
The ear lobe perforation service can be performed at pharmacies that have the specific piercing device or a sterile needle.	F	Article 78°, section II, of RDC n°44/2009.[1]
Pharmacies can sell serums and vaccines.	T	Article 7° of Law n° 13.021/2014.[3]
Theme: Dispensation of medication subject to special control.		

Questions	Answer (T/F*)	Reference
Preparations based on tramadol, associated with one or more components, should be dispensed through presentation and withholding of the special control prescription (which must be presented in two copies), regardless of the concentration.	T	Article 52° of Ordinance n° 344/1998; e List A2, Addendum 3°, of Ordinance n° 344/1998.[4]
Retinoic substances for topical use (e.g., tretinoin) must be sold by means of a special control prescription (which must be presented in two copies).	F	List C2, Addendum 2°, of Ordinance n° 344/1998. [4]
Sibutramine should be sold by withholding the special control prescription (which must be presented in two copies).	F	Article 2° of RDC n°52/2011.[5]
Diazepam 5 mg or 10 mg can be dispensed for up to 120 days of consumption, provided it is contained in a medical prescription.	F	Article 46° of Ordinance n° 344/1998. [4]
If the client uses fluoxetine 20 mg continuously and does not present a medical prescription, but has a scheduled medical appointment, it is permitted to sell the quantity of this medication until the date of the scheduled consultation.	F	Article 52°, paragraph 1°, of Ordinance n° 344/1998. [4]
The blue prescription notification is intended for dispensation of narcotic medication.	F	Article 1°, chapter 1°, of Ordinance n° 344/1998. [4]
Prescription notifications may contain more than one prescribed medication, provided that these medicinal products are from the same pharmacological group.	F	Article 35°, paragraph 7°, of Ordinance n° 344/1998. [4]
If the prescription of a medication under special control is not dated, the pharmacist is permitted to date the prescription with the date of dispensation.	F	Article 35°, paragraph 4°, of Ordinance n° 344/1998. E Article 36° of Ordinance n° 344/1998. [4]
It is prohibited to dispense diazepam 5 or 10 mg when prescribed by a dental surgeon.	F	Article 38° of Ordinance n° 344/1998. [4]
A medical prescription from another state in which paroxetine 20 mg is prescribed is permitted, provided that the pharmacist presents this prescription to the local health authority within a period of up to 72 hours.	T	Article 52°, paragraph 3°, of Ordinance n° 344/1998. [4]
Prescription of antiparkinson and anticonvulsant medications may contain enough for up to six months of treatment.	T	Article 59° of Ordinance n° 344/1998. [4]
The special control prescription can contain a maximum of three medications from the C1 list.	T	Article 57° of Ordinance n° 344/1998. [4]

Questions	Answer (T/F*)	Reference
The prescription of anabolic steroids may be valid for up to six months.	F	Article 59° of Ordinance n° 344/1998. [4]
Theme: Dispensation of generic medication.		
Analyze the following situation: During the dispensation of a generic medication, a customer asks the following question: "Why is the generic medication cheaper than the reference?" A possible answer would be: This medication is probably cheaper since the manufacturer has not invested in research to discover the active principle.	T	Article 3°, clause XXII e XXI, of Law n° 9787/1999.[6]
If the client requests, any pharmacy employee may substitute a reference (ethical) for a generic medication.	F	Clause VI, paragraph 2.1, of RDC n° 135/2003.[7]
Look at the following situation: "A customer arrives at the pharmacy and questions the effectiveness of generic medication, and you advise that generic medication does not have the same quality as the reference medication in most cases."	F	Article 3°, clause XXI, of Law n° 9787/1999. [6]
A similar medication may be replaced by a reference medication provided that bioequivalence tests have been carried out to prove their interchangeability.	T	Article 2° of RDC n° 58/2014. [8]
Theme: Dispensation of antimicrobials.		
The antimicrobial should be dispensed by retention of the second copy of the prescription, and delivery to the patient of the first copy stamped in the pharmacy.	T	Article 9° of RDC n° 20/2011.[9]
The prescription of antimicrobial medication is valid throughout the national territory up to 20 days after the date of issue.	F	Article 6° of RDC n° 20/2011. [9]
The prescription of antimicrobial medication should be made by a special two-copy prescription.	F	Article 5° of RDC n° 20/2011. [9]
There is no maximum limit on the amount of antimicrobials contained on a prescription.	T	Article 7° of RDC n° 20/2011. [9]
The sale of antimicrobials via telephone or the Internet is not permitted.	F	Article 11° of RDC n° 20/2011. [9]

Questions	Answer (T/F*)	Reference
It is permitted to contain other medication on the prescription of antimicrobials, provided that they are not subject to special control.	T	Article 7° of RDC n° 20/2011.[9]
The prescription of antimicrobials for continuous use may be valid for up to 90 days.	T	Article 8° of RDC n° 20/2011.[9]
Theme: Dispensation of medication exempt from medical prescription.		
The following classes of medications are permitted to be sold freely, i.e. without the presentation of a medical prescription: glucocorticoids, antihypertensives and oral antidiabetics.	F	Article 2° of RDC n° 138/2003.[10]
All anti-inflammatory medications are permitted to be sold without a prescription.	F	Article 2° of RDC n° 138/2003.[10]
Omeprazole (10 mg, 20 mg, 40 mg) can only be sold with a prescription.	T	Article 2° of RDC n° 138/2003.[10]
Nasal decongestants containing vasoconstrictors may be sold without a prescription.	F	Article 2° of RDC n° 138/2003.[10]
Theme: Dispensation of medication belonging to the Popular Pharmacy of Brazil program and/or used for the most prevalent diseases in Brazil.		
If a customer in the pharmacy starts treatment with metformin 850 mg and complains of diarrhea at the start of treatment, it is prudent to notify the industry and exchange for another batch of metformin 850 mg.	F	Fujoka, 2005.[11]
Glibenclamide 5 mg, when prescribed, should be taken half an hour before meals (breakfast, lunch and dinner) with a glass of water.	T	Chantal, 2014.[12]
A 25 mg/ml ferrous sulfate solution is best absorbed when administered with a glass of milk.	F	CANÇADO et al., 2010.[13]
If the client complains of stomach pain, it is prudent to indicate the administration of any medication with food.	F	CHANTAL, 2013.[12]
Captopril 25 mg, when prescribed, should be given one or two hours after meals.	T	DIPIRO, 2009.[14]
Hydrochlorothiazide 25 mg, when prescribed, may be taken after fasting or with food.	T	MICROMEDEX, 2015a.[15]

Questions	Answer (T/F*)	Reference
Simvastatin, when prescribed, may be taken on awakening or before sleeping.	F	Wallace, Chin, Rubin, 2003.[16]
After administration of inhaled corticosteroids the patient should wash the mouth immediately.	T	Dipiro, 2009.[14]
Patients using hydroxychloroquine should be advised that this medication may cause ocular side effects.	T	Latasiewicz, et al,2017.[17]
Propranolol may exacerbate the symptoms of asthma.	T	Morales, et al, 2017.[18]
Insulin in use may be kept at room temperature (15 °C to 30 °C) for up to 30 days, or under refrigeration (2 °C to 8 °C) for up to three months.	T	Grajower et al., 2003.[19]
The onset of the effect of fluoxetine 20 mg is usually noticed after two weeks of treatment, therefore the patient should be encouraged to continue treatment.	T	BRUNTON, 2012.[20]
The patient must be advised that carbamazepine may provide diplopia (perception of two images of a single object).	T	BARRETO, MASSABKI, 2010.[21]

*T: True; F: False.

References:

1. It provides for the sanitary control of the drug trade, drugs, pharmaceutical inputs and correlates, and other measures. Law n. 5991/1973. Brazilian Official Gazette; Section 1 p. 13049, December, 19 (Dez 19,1973.
2. Provides for Good Pharmaceutical Practices for sanitary control of the operation, dispensing and commercialization of products and the provision of pharmaceutical services in pharmacies and drugstores and provides other measures. Resolution n. 44, Aguste, 17. Brazilian Official Gazette (18 ago. 2009).
3. Provides for the exercise and supervision of pharmaceutical activities. Law n. 13021, Aguste, 8, 2014. Brazilian Official Gazette; Section 1, p.1 (11 ago 2014).

4. Approves the technical regulation on substances and medicinal products subject to special control. Order n. 344, May, 12, 1998. Brazilian Official Gazette; Section 1 p.3 (May, 15, 1998).
5. Suspend the Resolution RDC n. 52, October, 6, 2011, which provides for the prohibition of the use of the substances amfepramone, fenproporex and mazindol, their salts and isomers, as well as intermediates and measures to control the prescription and dispensation of medicinal products containing the substance sibutramine, its salts and isomers, as well as intermediates. Degree n. 273, September, 4. Brazilian Official Gazette; Section 1 p. 1 (Sep,5,2014).
6. Change the Law n. 6360, February, 23, September, 23, 1976, which provides for health surveillance, establishes the generic medicine, provides for the use of generic names in medicines and provides other measures. Law n. 9787, February, 10. Brazilian Official Gazette; Section 1 p. 1 (Feb 11, 1999).
7. Provides on Technical Regulation for Generic Medicines. Resolution RDC n. 135, May 29, 2003. Brazilian Official Gazette. (August 12,2003).
8. Provides for measures to be adopted at Anvisa by drug registration holders for the interchangeability of similar drugs with the reference product. Resolution n. 58, October 10, 2014. Brazilian Official Gazette. (Oct 13, 2014).
9. Provides for the control of medicines based on substances classified with antimicrobials, prescribed, isolated or in combination. Resolution RDC n. 20, May, 5, 2011. Brazilian Official Gazette; Section 1 p. 32 (May 9, 2011).
10. Provides for "Over the Counter" medicines. Resolution n. 138, May 19, 2033. Brazilian Official Gazette; Section 1, p. 32 (June 02, 2003).
11. Fujoka, R. L.et al. Efficacy, dose–response relations hip and safety of once-daily extended-release metformin (Glucophage XR) in type 2 diabetic patients with

inadequate glycemic control despite prior treatment with diet and exercise: results from two double-blind, placebo-controlled studies. Swaninkand M. Pans Diabetes, Obesityand Metabolism. 2005; n. 7:28–39.

12. Chantal S, Hazel E, Van Dorp F. Oxford Handbook of General Practice. Oxford Univerty Press; 3th ed, 2014.
13. Cançado, R. D.; Lobo, C.; Friedrich, J. R. Tratamento da anemia ferropriva com ferro por via oral. Rev. Bras. Hematol. Hemoter. 2010; v 32 (2): 114-120.
14. Dipiro J.; Talbert B.; Yee G. C.; Matzke G. R.; Wells B. G.; Posey L. M. (eds.). Pharmacotherapy: A Pathophysiologic Approach**,** 7th ed., New York, McGraw-Hill, 2008. p. 909-1203.
15. Micromedex, Hydrochlorothiazide, 2015b. Available from: http://www.micromedexsolutions.com/micromedex2/librarian/PFDefaultActionId/evidencexpert.DoIntegratedSearch. Access in Aug11, 2015: 04:35 pm.
16. Wallace A, Chinn D, Rubin G. Taking simvastatin in the morning compared with in the evening: randomised controlled trial. BMJ. 2003; 327 (7418):788.
17. Latasiewicz M, Gourier H, Yusuf IH, Luqmani R, Sharma SM, Downes SM. Hydroxychloroquine retinopathy: na emerging problem. Eye (Lond). 2017; 31(6): 972–976.
18. Morales D R, Lipworth B J, Donnan P T, Jackson C, Guthrie B. Respiratory effect of beta-blockers in people with asthma and cardiovascular disease: population-based nested case control study. BMC Medicine. 2017; 15:18. DOI 10.1186/s12916-017-0781-0.
19. Grayower, M. M.et al. How Long Should Insulin Be Used Once a Vial Is Started? Diabetes care. 2003; v. 26 (9): 2665 – 2669.

20. Brunton, L.L. The Pharmacological Basis of Therapeutics. McGraw-Hill, 12st ed. Rio de Janeiro: 2012.
21. Barreto B. C. S.; Massabki P. S. Adverse effects on the central nervous system by antiepileptic drugs in elderly. Rev Bras Clin Med. 2010; v. 8 (4): 344-349.
