# Supplementary material for: Development and validation of an instrument to measure the professional’s knowledge of dispensing medication (CDM-51) in community pharmacies
Source: PLoS One. 2020 Mar 3;15(3):e0229855. doi: 10.1371/journal.pone.0229855 (PMC7053717; doi:10.1371/journal.pone.0229855)
Supplement: S1 Data — (PDF) [file pone.0229855.s002.pdf]

Ribeirão Preto
28 de Janeiro, 2020

I, John Carpenter, declare that I am a native English speaker (British) and I have made a revision of the text:

DEVELOPMENT AND VALIDATION OF AN INSTRUMENT TO MEASURE THE PROFESSIONAL'S KNOWLEDGE OF DISPENSING MEDICATION (CDM-51) IN COMMUNITY PHARMACIES

I believe the standard of English in the text is acceptable for publication in the journal Plos One.

John Carpenter
(djcingles.com.br CNPJ: 22.621.433/0001-05)

DJCINGLES
Serviços em Língua Inglesa
CNPJ 22.621.433/0001-05
djcingles.com.br
